# Study of the microstructure of brain white matter in medial temporal lobe epilepsy based on diffusion tensor imaging

**DOI:** 10.1002/brb3.2919

**Published:** 2023-03-07

**Authors:** Yiwei Zhang, Zhaoxi Liu, Wanchen Dou, Juan Wei, Yuelei Lv, Bo Hou, Hui You, Feng Feng

**Affiliations:** ^1^ Department of Radiology Peking University First Hospital Beijing China; ^2^ Department of Radiology Peking Union Medical College Hospital, Chinese Academy of Medical Sciences and Peking Union Medical College Beijing China; ^3^ Department of Neurosurgery Peking Union Medical College Hospital, Chinese Academy of Medical Sciences Beijing China; ^4^ GE Healthcare, MR Research China Beijing China; ^5^ Department of Radiology Beijing CHAO‐YANG Hospital, Capital Medical University Beijing China; ^6^ State Key Laboratory of Difficult, Severe and Rare Diseases, Peking Union Medical College Hospital Peking Union Medical College and Chinese Academy of Medical Sciences Beijing China

**Keywords:** asymmetry, DTI, mTLE, surgical outcome, white matter fiber tract

## Abstract

**Objectives:**

To compare the white matter (WM) asymmetry in left and right medial temporal lobe epilepsy (mTLE) with and without hippocampal sclerosis (HS+, HS–) and assess the correlation of preoperative asymmetry and the dynamics of WM fibers with surgical outcomes.

**Materials and methods:**

Preoperative MRI scans were collected from 58 mTLE patients (40 HS+, 18 HS–); 15 (11 HS+, 4 HS–) then underwent postoperative MRI scans. DTI parameters, including the fractional anisotropy (FA), mean diffusion coefficient (MD), axial diffusion coefficient (AD), and radial diffusion coefficient (RD), were extracted from 20 paired WM tracts by PANDA based on the JHU WM tractography atlas. The bilateral cerebral parameters and the pre‐ to postoperative changes in the DTI parameters of specific fiber tracts were compared. The asymmetry indexes (AIs) of paired fibers were also analyzed.

**Results:**

There were fewer asymmetrical WM fibers in HS– patients than in HS+ patients. The pattern of WM asymmetry differed between left and right mTLE patients. Differences in the FA AI of the inferior fronto‐occipital fasciculus and inferior longitudinal fasciculus (ILF) were found in left HS+ patients with different surgical outcomes. All mTLE patients exhibited decreases in FA and increases in MD and RD in specific ipsilateral WM fibers. In International League Against Epilepsy (ILAE) grade 1 patients, the MD values in the ipsilateral CGH increased over time, whereas the RD values in the ipsilateral ILF and the AD values in the ipsilateral ILF and UNC decreased. In ILAE grade 2–5 patients, the FA values in the ipsilateral cingulate gyrus part of the cingulum (CGC) increased over time.

**Conclusion:**

The WM tract asymmetry was more extensive in HS+ patients than in HS– patients. The preoperative WM fiber AIs in left HS+ patients may be useful for surgical prognosis. Additionally, pre‐ to postoperative changes in WM fibers may help predict surgical outcomes.

## Graphical abstract

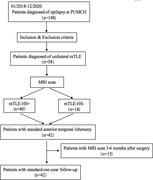

## 1 INTRODUCTION

Epilepsy is generally considered to be a chronic brain disease featuring repetitive, paroxysmal and transient central nervous system dysfunction caused by excessive discharge of brain neurons. Medial temporal lobe epilepsy (mTLE), as the most common drug‐refractory epilepsy, was once considered to be focal and confined to the gray matter of the temporal lobe; however, recent studies (Lin et al., 2020; Park et al., 2019) have found that the neural network involved in the generation, maintenance and propagation of seizures includes not only the cortical‐subcortical circuitry but also subcortical structures such as WM (white matter). As the connection between different parts of the cerebral cortex, WM constitutes the interactive network of the brain and serves the functions of transmission and feedback. Persistent seizure activity in patients with mTLE can lead to various forms of brain WM damage, such as reduced axonal density, axonal demyelination, and replacement of axons by glial cells (Rodríguez‐Cruces & Concha, 2015). Previous studies (Concha et al., 2009; Liu et al., 2014) have found that mTLE patients have extensive brain WM abnormalities inside and outside the temporal lobe. As the brain structural and functional changes related to mTLE have come to be understood, the concept of mTLE as a neural network disorder disease has been widely accepted.

Diffusion tensor imaging (DTI), as a noninvasive technique for evaluating the microstructure integrity of WM in vivo based on the measurement of the limited diffusion of water molecules, has been widely used in the analysis of WM defects in mTLE. The four main parameters of DTI possess different meanings. Fractional anisotropy (FA) is considered to reflect the fiber density, axon diameter, and myelination of WM of the brain. A decrease in FA value is usually considered to represent reduced integrity of WM fibers. The mean diffusion coefficient (MD) measures the degree of diffusion of water molecules that have nothing to do with the directionality. When the cell membrane, myelin, and other diffusion barriers are damaged, the MD value usually increases. The axial diffusion coefficient (AD), also known as the longitudinal diffusion coefficient, mainly reflects the diffusion coefficient along the main axis and is considered to be related to the integrity of the axon. The radial diffusion coefficient (RD) mainly reflects the diffusion coefficient perpendicular to the main axis and is considered to be related to changes in the myelin sheath. Studies (Kemmotsu et al., 2011) have shown that WM damage in mTLE patients mainly occurs on the side ipsilateral to the epileptic focus; compared with right mTLE patients, left mTLE patients have shown an increased extent of WM fiber bundle abnormalities, which contributes to epilepsy lateralization. Even mTLE patients who are negative for hippocampal sclerosis (HS) on the structural MRI can exhibit asymmetry in the bilateral WM microstructure (García‐Pallero et al., 2019; Yu et al., 2019), which is of important clinical significance for the preoperative diagnosis of laterality.

The brain WM of patients with epilepsy undergoes structural reorganization after surgery. Epilepsy surgery directly damages the WM by cutting the axons. WM damage caused by seizures may be reversible due to the termination of postoperative seizures (Yasuda et al., 2010). However, the prognosis of these injuries caused by chronic structural changes is still unclear. In addition, we cannot distinguish the cause of a patient's WM changes before surgery. Therefore, exploring the changes in brain WM before and after surgery is helpful for understanding the structural reorganization of the brain of patients with epilepsy and helps predict the prognosis of surgery, including seizures and other complications (Taylor et al., 2018).

DTI parameters were extracted by automatic processing in this study to prevent the intra‐tester error introduced in some previous studies by hand‐drawing regions of interest (ROIs) (Ahmadi et al., 2009). Many studies have focused on the asymmetry of bilateral WM in mTLE patients with HS (Zhao et al., 2019) or on the main DTI parameters (FA or MD values) (Li et al., 2019; Otte et al., 2012). Few studies have explored the relationship between preoperative WM asymmetry and surgical prognosis. The dynamic changes from before to after surgery have been even less studied, and the results of various DTI‐based studies are not consistent (Faber et al., 2013; McDonald et al., 2010; Winston et al., 2014).

Therefore, the main purpose of this study is to explore the asymmetrical features of the bilateral WM of left and right mTLE (including HS+ and HS–) patients based on DTI parameters (including FA, AD, RD, and MD) and compare the preoperative WM asymmetry characteristics of patients with different surgical prognoses. Additionally, we compared the changes in DTI parameters before and after surgery to explore the correlation between the dynamic changes in WM fibers and surgical outcomes.

## 2 MATERIALS AND METHODS

### 2.1 Patients

**FIGURE 1:**
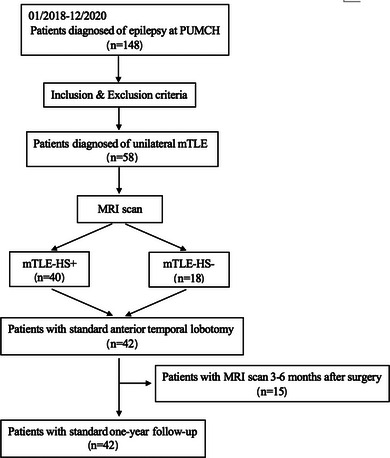
Flow chart of patient enrollment and classification.

This was a retrospective study, and subjects were enrolled for DTI analysis before and after surgical treatment (Figure 1).

#### 2.1.1 Preoperative patients

*Inclusion criteria*: (1) Prolonged video‐electroencephalography (vEEG) clearly captured the origin of the seizures. (2) Clinical semiology evaluation of epilepsy conformed to the characteristics of mTLE. (3) Interictal 18F‐fluorodeoxyglucose positron emission computed tomography (FDG‐PET) showed decreased metabolism in the unilateral medial temporal lobe.

*Exclusion criteria*: (1) TLE with bilateral seizures or difficulty in lateralization. (2) Symptomatic mTLE is caused by other reasons (such as tumors, vascular malformations, inflammation, etc.) at MRI. (3) There are other structural abnormalities outside the hippocampus at MRI (such as ischemic, hemorrhagic or traumatic abnormality, etc.). (4) Poor image quality caused by motion artifacts and other factors.

From January 2018 through December 2020, 58 consecutive unilateral mTLE patients were enrolled from the Neurology Unit of the Peking Union Medical College Hospital. All of the patients had frequent seizures and drug‐refractory epilepsy. The patients included in the study were divided into two subgroups, namely, the HS+ group and the HS– group, according to the presence or absence of signs of HS on MRI (hippocampal atrophy, signal changes, and loss of internal structure of the hippocampus). Two experienced neuroradiologists (YH with 15 years and FF with 25 years of experience) conducted the assessments together while blinded to the clinical information. Of the 58 patients included in this study (Table 1), the mean age was 29.2 ± 8.2 years, and the group comprised 29 women (50%) and 29 men (50%); 30 patients had left mTLE, and 28 had right mTLE; 40 cases had definite unilateral HS signs (HS+ group), and 18 had no obvious HS signs on MRI (HS– group). Forty healthy controls (HC) were also enrolled in the study (mean age: 27.0 ± 6.7 years; 20 males and 20 females) with no MRI‐identified structural abnormalities and no family history of neuropsychiatric disorders. The study was approved by the Institutional Review Board of Peking Union Medical College Hospital, and written informed consent was obtained from all subjects.

**TABLE 1:** Demographic characteristics and clinical data of enrolled patients and controls

	HS+ (*n* = 40)	HS– (*n* = 18)	HC (*n* = 40)	*p* Value
Gender (F/M)	16/24	13/5	20/20	.0759^a^
Age^b^	28.4 ± 8.8	31.1 ± 6.5	27.0 ± 6.7	.1691^c^
Age of onset^b^	15.9 ± 7.9	18.9 ± 6.3	/	.1637^d^
Disease duration^b^	12.4 ± 9.1	12.2 ± 8.0	/	.9282^d^
Lateralization (L/R)	22/18	8/10	/	.4567^e^
Febrile convulsion (±)	21/19	1/17	/	.0009^a^

^a^ Fisher's exact test.

^b^ Mean ± standard deviation.

^c^ ANOVA.

^d^ Independent‐sample *t*‐test.

^e^ Chi‐square test.

#### 2.1.2 Postoperative patients

Among the 58 patients, only 42 underwent standard surgical anterior temporal lobotomy and standard postoperative follow‐up for at least one year. An incision was made approximately 1 cm vertically into the temporal horn of the lateral ventricle, and approximately 5 cm from the middle temporal gyrus to the temporal pole, the anterior temporal lobe was disconnected from other brain tissue in the standard surgical anterior temporal lobotomy.

The patients’ surgical prognosis was classified according to the definitions given by the International League Against Epilepsy (ILAE) (Wieser et al., 2001). Patients were divided into two groups according to the presence of seizures after surgery. Seizure freedom was defined as an ILAE score of 1 (ILAE 1), and persistent postoperative seizures were defined as ILAE 2−6. Of the 42 patients, 28 patients (66.7%) had no seizures after surgery (ILAE 1), 14 patients (33.3%) still had different degrees of seizures after the operation (ILAE 2−5), and no patient had a postoperative prognosis grade of ILAE 6. MRI scans were acquired approximately 3−6 months after the operation in only 15 patients. The mean age of the 15 patients was 26.3 ± 8.2 years; this sample had 9 females (60%) and 6 males (40%). Of these patients, 5 (33.3%) had left mTLE, and 10 (66.7%) had right mTLE. There were 11 cases (73.3%) with HS and 4 cases (26.7%) without HS.

### 2.2 MRI acquisition

MRI scans included pre‐ and postoperative image acquisition. MRI scans of all patients were collected at least 48 h after the seizure. Postoperative MRI scans were acquired approximately 3−6 months after the surgery.

All patients underwent MRI scans on a 3.0 T scanner (Discovery MR750, GE Healthcare, Milwaukee, WI, USA) with a 32‐channel head coil. 3D T1‐weighted imaging (T1WI) (i.e., BRAin VOlume acquisition, or BRAVO) was acquired from each patient: the parameters were as follows: TR = 7.4 ms, TE = 2.8 ms, resolution = 1.0×1.0×1.0 mm, flip angle = 12°, TA = 6 min 35 s. DTI data were obtained in the axial plane by using a single‐shot diffusion‐weighted echo‐planar imaging pulse sequence with the following scanning protocol: TR = 5000 ms, TE = 62.3 ms, field of view (FOV) = 25.6×25.6 cm^2^, matrix dimensions = 128×128, slice thickness = 3 mm, number of slices = 51, number of diffusion gradient directions = 30, b = 0 and 1000 s/mm^2^, acceleration factor = 2, TA = 5 min 15 s.

### 2.3 DTI postprocessing

DTI data processing and analysis were performed on MATLAB R2018a (The MathWorks, Inc., USA). We used the brain diffusion image analysis software process package PANDA (Pipeline for Analyzing Brain Diffusion Images) (Cui et al., 2013) to extract DTI parameters. PANDA calculates the regional diffusion metrics (FA, MD, AD, and RD) by averaging the values within each region of the Johns Hopkins University (JHU) WM tractography atlas (Wakana et al., 2007).

### 2.4 Statistical analysis

We used SPSS (IBM SPSS Statistics, Version 23.0) for statistical analyses. According to epilepsy lateralization and the existence of HS sign on the MRI scans, patients were divided into four groups: the right mTLE group with HS (RHS+), the left mTLE group with HS (LHS+), the left mTLE group without HS (LHS–), and the right mTLE group without HS (RHS–). To eliminate the potential confounding factors of the innate asymmetry of the human brain WM, the Z value based on the average value of the HC group of the corresponding cerebral hemispheres was used to normalize all DTI parameters. The paired *t*‐test was used to compare the symmetry of the standardized DTI parameters in the ROI of the paired WM fiber tracts on the ipsilateral and contralateral cerebral hemispheres of the epileptic foci in each group. In the WM fiber tracts with significant differences in the paired ROIs of the bilateral cerebral hemispheres, the asymmetry index (AI) of the DTI parameters was further calculated (formulas (1) and (2)) to quantify the difference between the ipsilateral and contralateral sides of the cerebral hemispheres. The patients’ surgical prognosis was classified according to the definitions given by the International League Against Epilepsy (ILAE) (Wieser et al., 2001). Patients were divided into two groups according to the presence of seizures after surgery. Seizure freedom was defined as an ILAE score of 1 (ILAE 1), and persistent postoperative seizures were defined as ILAE 2−6. The independent‐samples *t*‐test was used to compare the differences in the AI of DTI parameters between the different surgical prognosis groups. The paired *t*‐test was used to compare the preoperative and postoperative differences in DTI parameters of specific WM fiber bundles in different surgical prognosis groups.

(1) $$\mathrm{AI}\left(\mathrm{left}\;\mathrm{mTLE}\right)=\frac{\left(\mathrm{contralateral}-\mathrm{ipsilateral}\right)+{\left(L-R\right)}_{\mathrm{HCmean}}}{\left(\mathrm{contralateral}+\mathrm{ipsilateral}\right)}$$

(2) $$\mathrm{AI}\left(\mathrm{right}\;\mathrm{mTLE}\right)=\frac{\left(\mathrm{contralateral}-\mathrm{ipsilateral}\right)-{\left(L-R\right)}_{\mathrm{HCmean}}}{\left(\mathrm{contralateral}+\mathrm{ipsilateral}\right)}$$

## 3 RESULTS

### 3.1 Asymmetry of the white matter microstructure of the whole brain

Table 2 shows white matter fiber bundles whose DTI parameters were asymmetrical in the four groups of patients (RHS+, LHS+, RHS–, LHS–) after normalization.

**TABLE 2:** WM fiber bundles with asymmetrical DTI parameters (FA, MD, AD, RD) in the four groups

DTI parameter	FA			MD			AD			RD		
	WM fiber	*t* Value	*p* Value	WM fiber	*t* Value	*p* Value	WM fiber	*t* Value	*p* Value	WM fiber	*t* Value	*p* Value
RHS+ (*n* = 18)	CGC	–2.565	.02	ATR	2.130	.048	ATR	2.633	.017	CGC	3.374	.004
				CST	2.806	.012	CST	5.175	<.001	CGH	3.440	.003
				CGC	3.238	.005						
	CGH	−2.651	.017	CGH	3.613	.002	CGH	2.486	.024	IFO	3.073	.007
				IFO	3.591	.002						
				ILF	4.132	.001	ILF	3.555	.002	ILF	3.128	.006
	UNC	−4.032	.001	UNC	9.522	<.001						
				SLF	2.950	.009	IFO	2.743	.014	UNC	7.891	<.001
LHS+ (*n* = 22)	CGH	–3.857	.001	CGH	6.055	<.001	ILF	2.097	.048	CGH	5.650	<.001
	IFO	−2.686	.014							IFO	3.116	.005
	ILF	−4.006	.001	IFO	3.241	.004				ILF	5.947	<.001
	SLF	−3.688	.001	ILF	4.999	<.001	CST	−3.740	.001	SLF	3.032	.006
	UNC	−3.583	.002	SLF	2.767	.012				UNC	3.644	.002
	SLFt	−2.447	.023						SLFt	2.576	.018
RHS– (*n* = 10)				CGC	3.737	.005	CST	2.617	.028	CGC	3.192	.011
				CST	2.574	.03						
LHS– (*n* = 8)	ATR	–3.600	.009	ILF	2.508	.041	UNC	2.971	.021	ILF	2.430	.045
	SLF	–2.565	.037	UNC	3.404	.011	CST	–3.850	.006	SLFt	2.780	.027

*t* > 0 ipsilateral side > contralateral side.

*t* < 0 ipsilateral side < contralateral side.

In the RHS+ group, the FA values were asymmetric in fibers including the cingulate gyrus part of the cingulum (CGC), the hippocampal part of the cingulum (CGH) and the uncinate fasciculus (UNC). The MD values were asymmetric in fibers including the anterior thalamic radiation (ATR), the corticospinal tract (CST), the CGC, the CGH, the inferior fronto‐occipital fasciculus (IFO), the inferior longitudinal fasciculus (ILF), the temporal component of the superior longitudinal fasciculus (SLFt) and the UNC. The AD values were asymmetric in fibers including the ATR, CST, CGH, ILF, and IFO. The RD values were asymmetric in fibers including the CGC, CGH, IFO, ILF, and UNC.

In patients with LHS+ status, the FA and RD values were asymmetric in the CGC, IFO, ILF, superior longitudinal fasciculus (SLF), UNC, and SLFt. The RD value of the ipsilateral side was higher than that of the contralateral side. The MD values were asymmetric in the CGH, IFO, ILF, and SLF. The AD value was asymmetric in the IFL.

In patients with RHS– status, no significant difference was found between the FA values of left and right fiber bundles. The MD values were asymmetric in the CST and CGC. The RD values were asymmetric in the CGC. In patients with LHS– status, the FA values were asymmetric in fibers including the ATR and SLF. The MD values were asymmetric in fibers including the ILF and UNC. The AD values were asymmetric in the UNC. The RD values were asymmetric in the ILF and SLFt.

In white matter fiber bundles with symmetrical DTI parameters of all four groups, the FA value of the ipsilateral side was lower than that of the contralateral side. The values of parameters including MD, AD, and RD on the ipsilateral side were higher than those on the contralateral side. The AD values were asymmetric in the CST in all four groups, with the values on the right side being higher than those on the left side.

### 3.2 Preoperative WM asymmetry and surgical prognosis

Of the 58 patients, 42 underwent standard anterior temporal lobotomy and standard postoperative follow‐up for at least one year. Twenty‐eight patients (66.7%) had no seizures after surgery (ILAE 1), 14 patients (33.3%) still had different degrees of seizures after the operation (ILAE 2−5), and no patient had a postoperative prognosis grade of ILAE 6 (Table 3).

**TABLE 3:** Proportion of patients who received standard temporal lobotomy and standard postoperative follow‐up in each group

	ILAE 1	ILAE 2−5	*p* Value
HS+	23 (74.2%)	8 (25.8%)	.0824^a^
HS–	5 (45.5%)	6 (54.5%)	
Right mTLE	13 (61.9%)	8 (38.1%)	.5127^a^
Left mTLE	15 (71.4%)	6 (28.6%)	

^a^ Chi‐square test.

**FIGURE 2:**
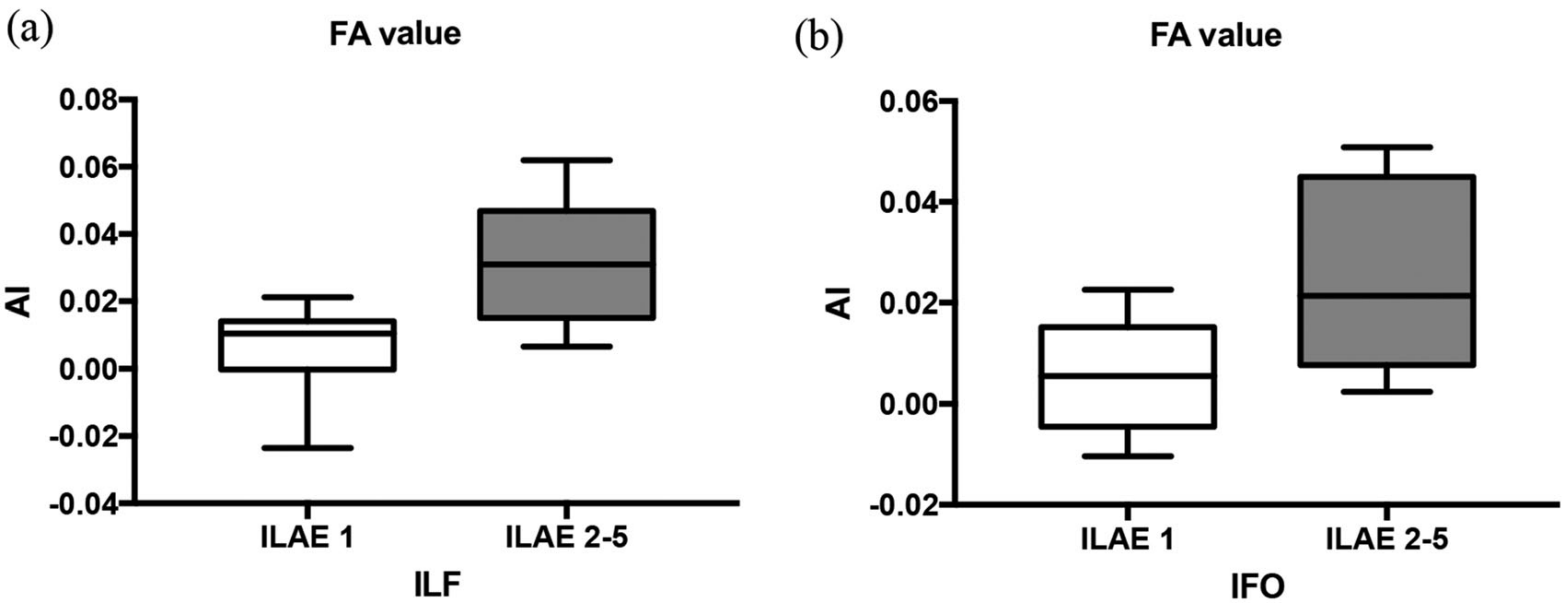
AIs of FA values in the IFO and ILF in LHS+ patients with different surgical prognoses.

In the white matter fiber bundle with a significant difference in paired bilateral ROIs, the AI of DTI parameters of the mTLE white matter fiber bundle was further calculated. In LHS+ patients, the preoperative AIs of FA values in the IFO (*p* = .023) and ILF (*p* = .009) in the ILAE 1 group were lower than those in the ILAE 2−5 group (Figure 2). There was no significant difference in DTI parameter AI in the other three groups.

### 3.3 Pre‐ to postoperative changes in WM and its correlation with surgical outcome

Among the 42 patients who underwent standard anterior temporal lobotomy and were followed up for at least 1 year, 15 patients underwent DTI scanning 3−6 months after the operation, of whom 11 patients (73.3%) had no seizures (ILAE 1), 4 patients (26.7%) still had seizures of different degrees (ILAE 2−5), and no patient had a postoperative prognosis grade of ILAE 6.

The FA values of ipsilateral CGH and UNC fiber bundles decreased after the operation compared with those before the operation in all patients. The MD values of the ipsilateral ILF, IFO, and UNC and the RD values of the ipsilateral CGH, IFO and UNC longitudinally increased. In addition, it was found that the MD value of the ipsilateral CGH, the RD value of the ipsilateral ILF, and the AD values of the ipsilateral ILF and UNC in patients without seizures (ILAE 1) increased longitudinally. In patients with different degrees of seizures (ILAE 2−5) after the operation, the FA value of the ipsilateral CGC was higher after the operation than before the operation; the AD value of the ipsilateral IFO increased longitudinally (Table 4). No significant difference was found in DTI parameters of contralateral white fibers before and after the operation.

**TABLE 4:** Pre‐ to postoperative changes in DTI parameters of ipsilateral fiber bundles

Surgical outcome	FA			MD			AD			RD		
	WM fiber	*t* Value	*p* Value	WM fiber	*t* Value	*p* Value	WM fiber	*t* Value	*p* Value	WM fiber	*t* Value	*p* Value
ILAE 1 (*n* = 11)	CGH↓ UNC↓	–8.212 −14.165	*p* < .001 *p* < .001	CGH↑ IFO↑ ILF↑ UNC↑	4.239 2.695 5.227 8.820	*p* = .002 *p* = .022 *p* < .001 *p* < .001	ILF↓ UNC↓	–7.454 −3.421	*p* < .001 *p* = .007	ILF↑ CGH↑ IFO↑ UNC↑	3.240 6.878 2.575 15.002	*p* = .009 *p* < .001 *p* = .028 *p* < .001
ILAE 2–5 (*n* = 4)	CGH↓ UNC↓ CGC↑	–19.524 −4.982 7.899	*p* < .001 *p* = .016 *p* = .004	IFO↑ ILF↑ UNC↑	6.233 3.506 4.302	*p* = .008 *p* = .039 *p* = .023	IFO↑	6.472	*p* = .007	CGH↑ IFO↑ UNC↑	5.601 3.709 5.373	*p* = .011 *p* = .034 *p* = .013

## 4 DISCUSSION

This study uses DTI technology and the JHU WM fiber tract atlas to extract and measure the characteristic values of DTI parameters of a total of 10 pairs of WM fiber tracts in bilateral cerebral hemispheres and explore the asymmetry of the whole brain WM microstructure in patients with left and right mTLE with and without HS. We tried to find the correlation between the features of and changes in DTI parameters and surgical outcome in patients with mTLE by comparing the AI of preoperative fiber bundle DTI parameters of different surgical prognosis groups, and the longitudinal DTI parameters changes of fiber bundle before and after surgery.

The Z value was calculated by using the average value of DTI parameters in the left and right hemispheres of the HC group to remove the inherent WM microstructure asymmetry of the human brain. We still found that mTLE patients had asymmetry of WM fiber bundles inside and outside the temporal lobe. The asymmetric characteristics of DTI parameters in the HS+ and HS– groups were similar. The FA value of the ipsilateral hemisphere was lower than that of the contralateral side, and the MD value of the ipsilateral hemisphere was higher than that of the contralateral side. Except for the CST fiber bundle, the AD and RD values of other fiber bundles showed asymmetry characteristics similar to those of MD. The decrease in the FA value of the ipsilateral hemisphere and the increase in the MD value led to asymmetry. The damage to the WM microstructure of the ipsilateral side by seizures may be more serious. For patients with mTLE, the surrounding WM fiber bundles may be affected by long‐term repetitive seizure discharges centered on the hippocampus, leading to pathological changes such as nerve fiber myelin degeneration, which is manifested as damage to the microstructure of the WM of the brain to varying degrees.

Although the asymmetry characteristics of the WM fiber bundles in the HS+ and HS– groups were similar, compared with the HS+ group, the number of asymmetric WM fiber bundles in the bilateral hemispheres in the HS– group was significantly reduced. The WM abnormalities of patients in the HS+ group were significantly more extensive, and these findings are also consistent with previous studies (Bernhardt et al., 2016; Liu et al., 2012; Scanlon et al., 2013). Another study found that compared with gender‐matched asymptomatic siblings and HCs, the FA values of the corpus callosum (CC), bilateral SLF, bilateral ILF, and left CST fiber bundles were significantly lower and the MD values of the lateral SLF and left CST were increased in HS– patients (Whelan et al., 2015). The abnormal fiber bundles involved (CGC, SLF, CST) are consistent with those that were identified our study, which indicates that HS– patients may also have extensive changes in WM fiber bundles inside and outside the temporal lobe.

This study found that the WM fiber tracts of the left and right medial temporal lobes had different asymmetric brain patterns in both the HS+ and HS– groups. Many recent studies have found that compared with right mTLE, WM abnormalities in left mTLE are more extensive (Pustina et al., 2015; Whelan et al., 2015). However, other studies (Corrêa et al., 2018; Liu et al., 2012) found that patients with right mTLE present more extensive changes than left mTLE patients, or that there is no significant difference in the extent of WM alterations between right and left mTLE patients. These results are controversial and further studies with larger sample sizes are needed. Studies have also found that when the ipsilateral hemisphere is consistent with the dominant hemisphere for language, mTLE patients exhibit more extensive WM microstructure abnormalities (Powell et al., 2007). The results of this study are only partially consistent with those reported in the literature. For HS+ patients, in terms of the asymmetry of FA and RD values, the number of abnormal fiber bundles in the LHS+ group (6 pairs each) was greater than the number in the RHS+ group (3 pairs and 5 pairs, respectively). However, in terms of the asymmetry of MD and AD values, the number of abnormal fiber bundles in the RHS+ group (8 pairs and 5 pairs, respectively) was greater than the number in the LHS+ group (4 pairs and 2 pairs). For HS– patients, in terms of the asymmetry of DTI parameters, the number of abnormal fiber bundles in the LHS– group (2 pairs each) was greater than or equal to the number in the RHS– group (1 or 2 pairs each). Studies have suggested that the left and right sides of mTLE are syndromes with different etiologies and pathologies from the start (Ahmadi et al., 2009). However, this has yet to be confirmed in a large number of studies.

There has been much evidence that in patients with mTLE, the WM fiber tracts are abnormal based on DTI imaging, especially in areas that are functionally or anatomically connected to the medial temporal lobe. Nevertheless, the relevance of these findings to the prognosis of surgery has not been fully established (Bonilha & Keller, 2015). It is undeniable that the causes of postoperative seizures in mTLE patients may be multifactorial and vary among different patients. However, most DTI‐based studies are dedicated to discovering imaging markers that may be related to the prognosis of surgery and providing new insights for mTLE. The study found that patients with a higher hippocampal AI value of MD before surgery have a better prognosis (Goncalves Pereira et al., 2006). In addition to the hippocampus, the relationship between extrahippocampal abnormalities and prognosis may also be very important. The abnormal diffusion of the ipsilateral posterior fornix and contralateral parahippocampal WM bundle of the epileptogenic focus before the operation indicates that there will be persistent seizures after the operation (Keller et al., 2017). Although these are only preliminary findings, there seems to be increasing evidence that WM is related to the clinical features and control of epilepsy. This study also preliminarily discussed the relationship between preoperative DTI parameter asymmetry and surgical prognosis. It was found that preoperative DTI parameter asymmetry has little significance in predicting postoperative epilepsy for patients who are RHS+ and HS– on MRI. The study of LHS+ patients found that the preoperative differences in fiber bundles were mainly concentrated in FA values of IFO and ILF. Compared with the group that was seizure free after surgery, the AI of these fiber bundles was increased in the group with poor prognosis. Previous studies have found that the WM microstructure abnormalities on the left mTLE are more extensive than those on the right, and the WM microstructure abnormalities in patients with HS are more extensive than those without HS. Therefore, the WM microstructure abnormalities in patients with left mTLE (LHS+) with HS may be more extensive than those in the other three groups. We boldly speculate that under the same surgical method and scope, the more serious the abnormal WM fibers outside the ipsilateral temporal lobe of LHS+ patients before surgery may indicate a worse prognosis after surgery.

In addition, this study further addressed the relationship between preoperative and postoperative longitudinal changes in fiber bundle DTI parameters and surgical prognosis. Compared with preoperative changes, the DTI parameters of CGH and UNC fiber bundles changed (FA value decreased, MD value increased, and RD value increased) on the ipsilateral side (i.e., surgical side) of all patients. It has been previously reported that the changes in these DTI parameters after the operation occurred in the area directly or indirectly connected to the surgical area, mostly occurring in the first year after the operation (Faber et al., 2013; Liu et al., 2013; Nguyen et al., 2011; Winston et al., 2014). After surgery directly cuts off the axon, Wallerian degeneration can lead to changes in the DTI parameters of the ipsilateral cingulate gyrus (CG) (Li et al., 2019). Previous literature also reported longitudinal changes in DTI parameters in ILF and IFO, which is consistent with this study (McDonald et al., 2010; Schoene‐Bake et al., 2009; Yogarajah et al., 2010). Although the fibers in these areas were not directly transected during surgery, they were more or less interrelated with the temporal lobe and were also affected by Wallerian degeneration after epilepsy surgery (Yogarajah et al., 2010). Compared with patients with poor prognosis (ILAE 2–5), those with better prognosis (ILAE 1) often have one or two more fiber bundles with longitudinal changes, but the change features of FA, MD and RD values are the same. The FA value of the CGC fiber bundle changed longitudinally only in patients with poor prognosis, but in contrast to those with good prognosis, it increased longitudinally after operation compared with that before operation. Many factors can affect the changes in FA value, such as the changes in intracellular and extracellular volume, the permeability of cell membrane, the cohesion of fibers, the loss of axons, the mechanical stretching and denaturation of cross fibers (Beaulieu, 2002). It is generally believed that the increase in the FA value is mainly related to the plasticity of the postoperative structure, not only in the language network but also in the memory system (Eacott & Gaffan, 2005; Nguyen et al., 2011). In this study, the longitudinal increase in the FA value of CGC may indicate that CGC may not be affected by Waller's degeneration but reconstructed after surgery in patents with poor surgical prognosis. This may have certain suggestive significance in predicting postoperative seizures. Previous literature has reported (Li et al., 2019) longitudinal changes in WM fibers in the contralateral (nonsurgical side) brain area. This may suggest that the contralateral cerebral hemisphere may also play a very important role in postoperative recovery. However, we did not find extensive longitudinal changes in DTI parameters on the contralateral (nonsurgical) side. This may be due to the limited recovery ability of the WM fibers within 3 to 6 months after surgery. Our study is only a very preliminary one. It is hoped that the diffusion change model based on DTI can have positive predictive value in the control, recurrence, cognitive improvement, or deterioration of epilepsy in the future.

There are several limitations in this study. First, the sample size was relatively small, especially for the collection of cases for HS– patients, which may be related to the fact that the prevalence of the HS– group itself was lower than that of the HS + group (accounting for only approximately 30% of mTLE (Muhlhofer et al., 2017)). Second, patients underwent anterior temporal lobotomy rather than lobectomy. We were unable to obtain histopathological outcomes of the hippocampus. Third, the cognitive function of patients was not evaluated in this study. Some cognitive functions, such as language, have been observed to be related to brain asymmetry, which may become an influencing factor. Additionally, DTI scans were acquired in a relatively short time frame (3–6 months after surgery) in this study due to some objective conditions, such as the compliance of patients for return visits. Studies focused on changes in WM based on DTI scans after a long time (at least one year after surgery) or the contrast between the changes in WM in long and short time frames need to be conducted in the future.

## 5 CONCLUSION

The asymmetry of WM fiber bundles in the bilateral cerebral hemispheres is more extensive in mTLE patients with HS than in patients without HS. mTLE had a more serious effect on the WM fiber bundles of the ipsilateral cerebral hemisphere than on those of the contralateral hemisphere. The preoperative WM fiber bundle AI of left HS+ patients may have some value for predicting patients’ postsurgical prognosis. In addition, the longitudinal changes in WM fiber bundles before and after surgery may provide further clues for predicting surgical prognosis.

## AUTHOR CONTRIBUTIONS

Yiwei Zhang was responsible for writing the manuscript and for analyzing and interpreting the data. Zhaoxi Liu helped collecting MRI data from the patients. Wanchen Dou helped with patient enrollment. Juan Wei was responsible for the postprocessing of the DTI data. Yuelei Lv helped with the patient enrollment. Hui You helped with design of the work and interpreting the images. Feng Feng explored the effect of asymmetrical brain features on lateralization of epileptic activity in temporal lobe epilepsy with and without hippocampal sclerosis.

## CONFLICT OF INTEREST STATEMENT

One of the authors, Wei Juan, works for GE Healthcare. The other authors have no conflicts of interest to disclose.

### PEER REVIEW

The peer review history for this article is available at https://publons.com/publon/10.1002/brb3.2919.

## Data Availability

The data that support the findings of this study are available from the corresponding author upon reasonable request. The data are not publicly available due to privacy or ethical restrictions.
